# circRNAprofiler: an R-based computational framework for the downstream analysis of circular RNAs

**DOI:** 10.1186/s12859-020-3500-3

**Published:** 2020-04-29

**Authors:** Simona Aufiero, Yolan J. Reckman, Anke J. Tijsen, Yigal M. Pinto, Esther E. Creemers

**Affiliations:** 1 Department of Experimental Cardiology, Amsterdam UMC, location AMC, Amsterdam, The Netherlands; 2 Department of Clinical Epidemiology, Biostatistics and Bioinformatics, Amsterdam UMC, Location AMC, Amsterdam, The Netherlands

**Keywords:** circRNA, R-package, Annotation, Differential expression analysis, Sequence analysis, Functional prediction

## Abstract

**Background:**

Circular RNAs (circRNAs) are a newly appreciated class of non-coding RNA molecules. Numerous tools have been developed for the detection of circRNAs, however computational tools to perform downstream functional analysis of circRNAs are scarce.

**Results:**

We present circRNAprofiler, an R-based computational framework that runs after circRNAs have been identified. It allows to combine circRNAs detected by multiple publicly available annotation-based circRNA detection tools and to analyze their expression, genomic context, evolutionary conservation, biogenesis and putative functions.

**Conclusions:**

Overall, the circRNA analysis workflow implemented by circRNAprofiler is highly automated and customizable, and the results of the analyses can be used as starting point for further investigation in the role of specific circRNAs in any physiological or pathological condition.

## Background

Next-generation sequencing has revealed the expression of thousands of circular RNAs (circRNAs) in eukaryotic cells. At this time, no *general* function of circRNAs has been uncovered, but several lines of evidence indicate that circRNAs can act as microRNA sponges, sequester RNA binding proteins (RBPs), regulate transcription or provide a template for protein translation [1]. CircRNAs are formed during pre-mRNA splicing, by a back-splicing event of one or two exons. They contain a unique exon-exon junction (also called back-splice junction (BSJ)), not present in the linear host transcript. Bioinformatic tools take advantage of this unique feature and use the BSJ-spanning reads to identify circRNAs from RNA sequencing data. Over a dozen of these circRNA detection tools have been developed, each differing in specificity, sensitivity, and criteria to define a circRNA. Due to these differences the use of multiple tools for circRNA detection is recommended to have the most accurate circRNA prediction.

With increasing numbers of circRNAs detected by these bioinformatics tools, the need for pipelines to analyze expression and putative functions of circRNAs became imperative. This led to the development of tools that cover different aspects of in silico circRNA analysis, from prediction to first functional insights (e.g. Circtools, CircPro and CircInteractome) [2–4]. These tools have specific strengths and limitations. For instance, Circtools combines circRNA data with positional data from eCLIP experiments to explore RBP sponge functions of circRNAs [2]. Circtools is also useful to model changes in circRNA expression relative to that of the host gene. CircPro enables users to discover circRNAs with protein-coding potential [3]. Limitations include restrictions on the use of specific aligners to map reads, installation of additional software, and compatibility with only one circRNA detection tool.

Here we present circRNAprofiler, an R-based framework that only requires an R-installation and offers 15 modules for a comprehensive in silico analysis of circRNAs. This computational framework allows to combine and analyze circRNAs detected by multiple publicly available annotation-based circRNA detection tools. It covers different aspects of circRNA analysis ranging from differential expression analysis, genomic context, evolutionary conservation, biogenesis to functional analysis. The pipeline used by circRNAprofiler is highly automated and customizable. Furthermore, circRNAprofiler includes additional functions for data visualization to facilitate interpretation of the results.

### Implementation

**Fig. 1:**
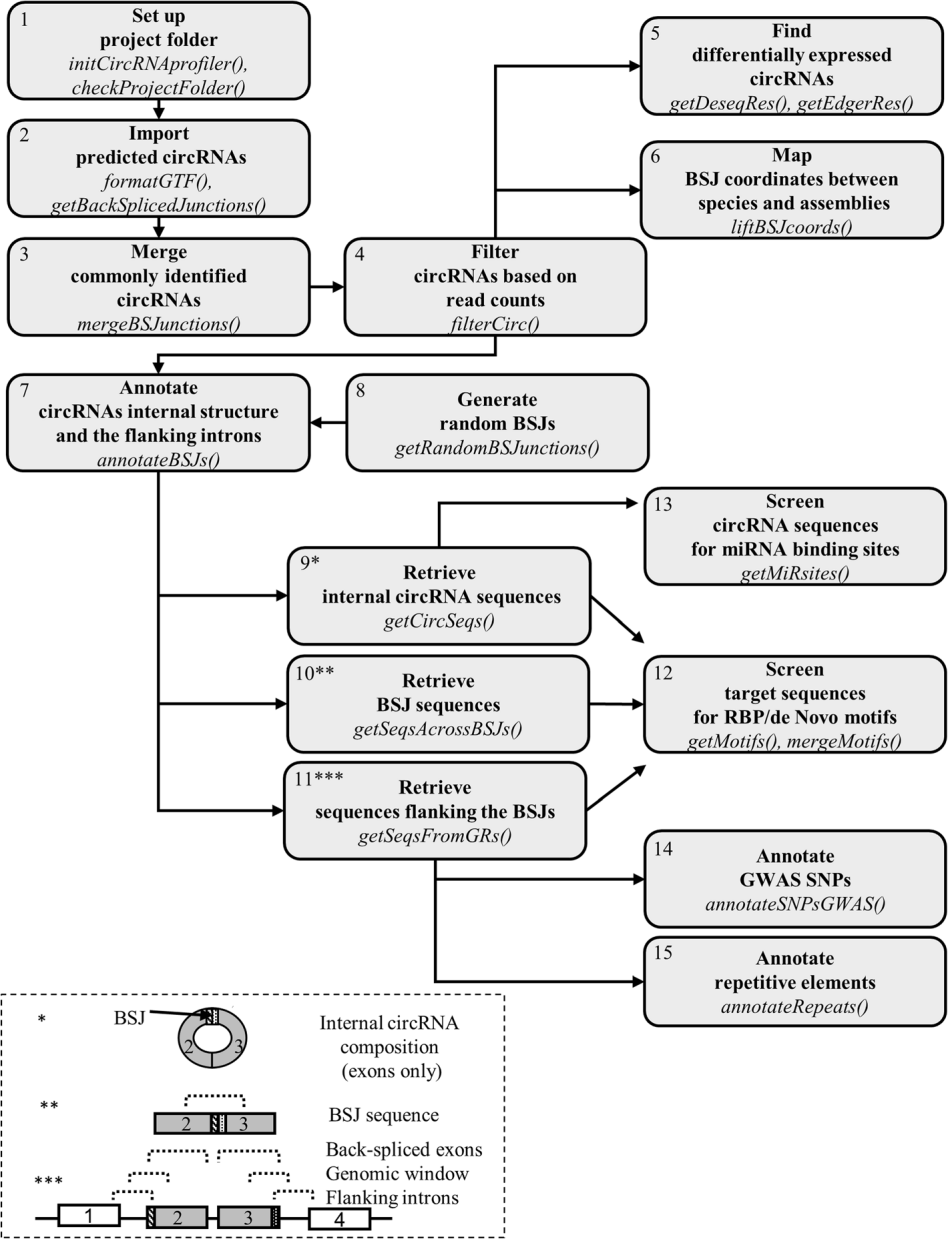
Schematic representation of the circRNA analysis workflow implemented by circRNAprofiler. The grey boxes represent the 15 modules described in the text with the main R-functions reported in italics. The different type of sequences that can be selected are depicted in the dashed box. BSJ, Back-Spliced Junction

The workflow of circRNAprofiler is shown in Fig. 1. Detailed information on each module can be found in the package vignettes. Following, we only give a brief overview of the circRNA analysis workflow implemented by circRNAprofiler.

In *module 1*, the project folder is set up to start the analysis. The project folder contains project files and the circRNA prediction results. In detail, project files include the genome-annotation file, the file containing information about the experimental design and optional files containing user specifications that are used to customize the analysis to execute.

In *module 2*, circRNAs detected by one or more of the annotation-based circRNA detection tools MapSplice2 [5], NCLscan [6], CircMarker [7], CircExplorer2 [8], KNIFE [9], UROBORUS [10] are imported in R working environment.

In *module 3*, results of different circRNA detection tools are merged to create a unique data set ready to enter the downstream analyses.

In *module 4*, circRNAs are filtered on user-defined read counts to eliminate false positives.

In *module 5*, a pairwise comparison is executed to identify differentially expressed circRNAs.

The R Bioconductor packages DESeq2 [11] or EdgeR [12], which implement a beta-binomial model are used to model changes in circRNA expression.

*Module 6* is used for conservation analysis of BSJ-coordinates across different species. The liftOver [13] utility from UCSC is used for this purpose.

In *module 7*, the internal exonic composition of circRNAs, as well as their flanking introns, are annotated. The genomic features are extracted from the user-provided gene annotations.

*Module 8* offers the possibility to incorporate a set of random BSJs as an independent dataset. This may, for instance, be used for enrichment analysis in microRNA/RBP binding sites in a set of circRNAs.

In *Modules 9*, internal circRNA sequences (i.e. the exonic sequences between the BSJs) are extracted.

In *Module 10*, back-spliced junction sequences only are extracted.

In *Module 11*, the user can extract sequences flanking BSJs. These are: i) sequences of the introns flanking BSJs, ii) sequences from a user-defined genomic window surrounding the back-spliced junctions and iii) sequences of the back-spliced exons only.

Sequences extracted with *Module 9–11* can enter the downstream screenings.

In *module 12*, sequences from module 9–11 are screened for the presence of RBP motifs (from ATtRACT or MEME databases), de novo motifs or motifs specified by the user.

In *module 13*, circRNA sequences are screened for miRNA binding sites. The user can select stringency criteria for miRNA binding prediction.

In *module 14*, SNPs of genome-wide association studies (GWAS catalog [14]) are annotated in the selected regions flanking BSJs to identify SNPs affecting circRNAs biogenesis.

In *module 15*, repetitive elements from the RepeatMasker database [15] are annotated in regions flanking the BSJs to investigate circRNAs biogenesis.

## Results

As a practical example, total RNA-sequencing data from human control hearts, dilated cardiomyopathy (DCM) tissues and hypertrophic cardiomyopathy (HCM) tissues (*n* = 3/group) were analyzed with 3 different circRNA detection tools: CircMarker [7], MapSplice2 [5] and NCLscan [6] (see Additional File 1 for detailed method).

### Analysis of the identified circRNAs – circRNAprofiler (development version 1.1.16)

After circRNA detection, the identified BSJ-spanning reads were analyzed with circRNAprofiler. The following experimental workflow is an example, but the pipeline used by circRNAprofiler is highly automated and customizable (i.e. different settings can be applied by the user).

### Module 1 - set up project folder

First, the project folder is set up, which contains project files and the circRNA prediction results from CircMarker (cm), MapSplice2 (ms), and NCLscan (ns).

### Module 2 - import predicted circRNAs

Files containing the detected circRNAs are imported in the R working environment. MapSplice2 predicted 7499 putative circRNAs, NCLscan identified 16,771 putative circRNAs, and CircMarker predicted 39,251 different circRNAs to be expressed in the heart samples (Additional File 1, Figure S1A; Additional File 2, sheet 1).

### Module 3 - merge commonly identified circRNAs

The detected circRNAs were grouped, and we found that a total of 41,558 unique circRNAs were identified by the three detection tools (Additional File 2, sheet 2). Of these, a small subset of circRNAs (186 circRNAs) were derived from the antisense strand of the reported gene (i.e. antisense circRNAs, see Additional File 2, sheet 3). In that case, the strand from which the circRNA arises (i.e. reported in the prediction results) is different from the strand of which the gene is transcribed (i.e. reported in the genome annotation file). Interestingly, the back-spliced junction coordinates overlap with exon coordinates of the reported gene. This might be explained by technical artifacts or by the presence of a gene transcribed from the opposite strand that is not annotated. Furthermore, the expression of this subset of circRNAs is rather low and rather variable across samples. Due to the ambiguous nature of these predictions, the detected antisense circRNAs are excluded from the circRNA analysis pipeline.

### Module 4 - filter circRNAs

CircRNA prediction results were filtered based on read counts (≥5 present in at least 3 samples of 1 condition) and found that 1458 circRNAs survived the filtering step with the majority of them (73%) detected by all 3 prediction tools (Additional File 1, Figure S1B; Additional File 2, sheet 4). The Na^+^/Ca^2+^ exchanger gene, Solute Carrier Family 8 Member A1 (SLC8A1) is considered to be the most abundantly expressed circRNA in the human heart [16–18]. Our analysis, however, revealed that CircMarker detected 3 circRNAs arising from the genes SYCP2, AL132709, and LGMN, which were predicted to be expressed approximately to the same level as SLC8A1, or even higher in case of circSYCP2 (Additional File 2, sheet 4). Since this high level of circSYCP2, circAL132709 and circLGMN expression was surprising to us, we verified their expression, together with circSLC8A1 experimentally, by RT-PCR in 4 human heart samples, with and without RNAse R treatment, using our previously described methods [16] (Additional File 1, Figure S1C). We did not detect a clear and specific amplicon at 35 and 40 PCR cycles for these three circRNAs, while circSLC8A1 was readily detectable already at 28 PCR cycles. This suggests that these 3 circRNAs are not as highly expressed as circSLC8A1 in the heart. However, we can not rule out that problems with primer efficiencies or design might underlie the lack of amplification for circAL132709 and circLGMN. To search for a positive control for our primers, we tested the same primers in an extensive human tissue panel of 22 different organs (Clontech, Cat No. 636643). We detected circAL132709 in adult liver and circSYCP2 faintly in fetal liver at 40 PCR cycles (Additional File 1, Figure S1D), indicating that the primers for circSYCP2 and circAL132709 are able to detect the specified circRNA. Therefore, we conclude that circSYCP2 and circAL132709, which are predicted to be highly expressed by CircMarker, are most likely not, or only lowly expressed in the heart. Overall, this suggests to us that the underlying algorithm of CircMarker is more prone to false-positive predictions and the results should be handled carefully.

### Module 5 - differential circRNA expression analysis

Differential expression analysis on the 1458 filtered circRNAs revealed that 9 out of 1458 circRNAs were differentially expressed in DCM patients compared to control samples, 97 circRNAs were differently expressed in HCM patients compared to control samples, and 53 circRNAs were differentially expressed in HCM compared to DCM patients (Additional File 1, Figure S2A-C; Additional File 2, sheet 5–7). The helper function for the R Bioconductor package DESeq2 [11] was used. As a cut-off, we used an absolute log2FC = 1 and an adjusted *p*-value≤0.05. Together with TTN circRNAs known to be deregulated in DCM and HCM patients [16] we found an additional single-exon circRNA to be highly expressed and dysregulated in DCM compared to control samples. This circRNA arises from the ALPK2 gene, which is reported to be highly expressed in the heart (GTEx portal).

### Module 6 - conservation analysis of the BSJ coordinates

We mapped the BSJ coordinates of the 1458 filtered circRNAs to mouse genome coordinates and found that approximately 50% of the human BSJ coordinates were conserved to mice (Additional File 2, sheet 8).

### Module 7 and 8 - analysis of the genomic context of circRNAs

Structural characterization of the 1458 filtered circRNAs revealed that introns flanking the predicted BSJs are longer than introns flanking randomly generated back-spliced exons (Additional File 1, Figure S2D). This is commonly observed by others as well [19]. Furthermore, we did not find a difference in the length of the predicted back-spliced exons compared to the set of randomly generated back-spliced exons (Additional File 1, Figure S2E). Of note, the set of randomly generated back-spliced exons is a set of exons, that are normally not back-spliced. This set is solely created to be used for comparative purposes in the different analyses (e.g., exon-length, miRNA binding, RBP binding). We analyzed the number of different circRNAs per host gene and found that approximately half of the circRNAs-producing genes (676 genes) produced a single circRNA, while the other half generated between 2 to 46 different circRNAs. The two genes that produced the highest number of circRNAs were TTN and RyR2, which generated a total of 46 and 29 putative circRNAs, respectively (Additional File 1, Figure S2F). Most back-spliced exons were predicted to comprise between 1 and 6 exons (Additional File 1, Figure S2G).

### Module 9, 10 and 11 - retrieval of internal circRNA sequences, BSJ sequences and sequences flanking the BSJ

In these modules, the user can extract sequences of interest for further downstream analysis. The package is designed to be able to analyze one or multiple circRNAs in the same run. **We** selected the single-exon circRNA arising from the ALPK2 gene for further analysis because we found it to be differentially expressed in DCM hearts compared to control hearts. Therefore, we retrieved the circRNA sequence of the gene ALPK2 (module 9), as well as the sequences surrounding the BSJ (module 11) of ALPK2.

### Module 12 - RBP analysis on the internal circRNA sequence and on sequences surrounding the BSJs

CircRNAprofiler can be used to analyze RBP motifs on the internal circRNA sequences. This analysis is useful to investigate whether the circRNA(s) in question is/are likely to bind or scavenge certain RBPs. RBP analysis of the circALPK2 exonic sequence revealed multiple enriched RBP motifs compared to the remaining subset of 1457 filtered circRNAs (Additional File 1, Figure S3A; Additional File 3, sheet 1–3).

To investigate circRNAs biogenesis, it may be useful to assess putative RBP binding sites in sequences flanking the BSJ. Therefore, we extracted sequences from a defined genomic window surrounding the predicted BSJ of circALPK2 (module 11) and analyzed these sequences for the presence of RBP motifs (default settings). A subset of 200 nucleotides were taken from the flanking introns and 10 nucleotides from the back-spliced exon. Interestingly, we found a ~ 4-fold enrichment in the introns surrounding circALPK2 for RBM24, a heart and skeletal muscle-specific splicing factor [20] (Additional File 1, Figure S3B; Additional File 3, sheet 4–6) compared to the remaining subset of 1457 filtered circRNAs. A similar result was observed when this circRNA was compared to a subset of sequences flanking randomly generated BSJs (data not shown). This suggests that RBM24 may be involved in the biogenesis of this particular circRNA.

### Module 13 - miRNA binding site analysis on the internal circRNA sequence

Next, we analyzed the internal circRNA sequence (retrieved from module 9) of circALPK2 for the presence of potential miRNA binding sites. The miRNA sequences we analyzed were downloaded from a study in which miRNA deep sequencing was performed on human left atria (LA) and ventricles (LV) under normal physiologic conditions [21]. From this study, we filtered the miRNAs based on their expression level in the LV (average expression of at least 10 reads in the 4 control samples). A subset of 361 miRNAs survived the filtering step and were used in our miRNA binding site analysis. As cut-off, we used ≥6 total matches between the seed region of the miRNAs and the seed site in the target sequence of which at least 5 are canonical Watson-Crick matches and 1 can be a non-canonical match (G:U). With this setting, we found several potential miRNAs binding sites within ALPK2 circRNA sequence, including a total of 48 putative sites for miR-130b-5p (Additional File 1, Figure S3C; Additional File 4).

### Module 14 - GWAS SNPs analysis on the intron flanking BSJs

We analyzed flanking introns (retrieved from module 11) of the 1458 filtered circRNAs for the presence of diseased-associated GWAS SNPs that may affect circRNAs biogenesis. In our analysis, we only considered GWAS SNPs associated with cardiac and vascular traits reported in the GWAS Catalog [14]. We found that the 241 GWAS SNPs are located within introns flanking the predicted circRNAs (Additional File 5). A further step could be to assess whether the risk allele is present in the genome of the analyzed samples (control and diseased patients). The presence of the risk allele, coupled with differential expression of the corresponding circRNAs, might be the first step to shed light on whether these SNPs might affect circRNAs biogenesis.

### Module 15 - repetitive elements analysis on the intron flanking BSJs

Finally, analysis of the introns flanking the predicted BSJs of the 1458 filtered circRNAs reveals that approximately half of them contain complementary Alu repeats (Additional File 6). The presence of complementary Alu repeats in introns is a known mechanism in the formation of circRNAs [22].

## Conclusion

circRNAprofiler provides an R-based framework that allows to combine circRNAs detected by multiple annotation-based detection tools and to perform structural and functional circRNA analysis. The results of all the aforementioned analyses can serve as starting point for further investigation in the role of specific circRNAs in heart disease. Above all, these analyses described here provides an example of how this R-package can be used in the study of circRNA expression, function, or biogenesis of any physiological or pathological entity. We intend to develop additional functions in the future to provide a comprehensive and up-to-date framework for the characterization of circRNAs.

### Availability and requirements

Project name: circRNAprofiler

Project home page: https://github.com/Aufiero/circRNAprofiler.

Operating system(s): Platform independent

Programming language: R

Other requirements: R v 3.6.0 or higher

License: GPL

Any restrictions to use by non-academics: No limitations.

## Supplementary information

**Additional file 1.** Supplementary methods and Figures. This file contains supplementary methods and figures.

**Additional file 2.** circRNAs detected in human hearts. This file contains circRNAs detected in human control hearts (C1, C2, C3), dilated cardiomyopathy (D1, D2, D3) tissues and hypertrophic cardiomyopathy (H1, H2, H3) tissues with 3 different circRNA detection tools: CircMarker (cm), MapSplice2 (ms) and NCLscan (ns). Results of the circRNA differential expression analysis. Human circRNAs conserved to mouse.

**Additional file 3.** RBP analysis. This file contains the results of the RBP analysis in the ALPK2 circRNA sequence and in the region flanking the BSJ of circALKP2.

**Additional file 4.** miRNA binding sites analysis. This file contains the results of the miRNA binding sites analysis in the ALPK2 circRNA sequence.

**Additional file 5.** GWAS SNPs analysis. This file contains the results of GWAS SNPs analysis on the intron flanking BSJs.

**Additional file 6.** Repetitive elements analysis. This file contains the results of the repetitive elements analysis on the intron flanking BSJs.

## Data Availability

Raw RNA sequencing data are available at NCBI BioProject accession number PRJNA533243. All data generated or analyzed during this study are included in this published article (and its supplementary information files).

## Abbreviations

CircRNA: Circular RNA
DCM: Dilated cardiomyopathy
HCM: Hypertrophic cardiomyopathy
RBM20: RNA-binding motif protein 20
RBM24: RNA-binding motif protein 24
RBP: RNA binding proteins
BSJ: Back-splice junction
miRNA: microRNA
SNPs: Single-nucleotide polymorphism
GWAS: Genome-wide association study
