# Supplementary material for: circRNAprofiler: an R-based computational framework for the downstream analysis of circular RNAs
Source: BMC Bioinformatics. 2020 Apr 29;21:164. doi: 10.1186/s12859-020-3500-3 (PMC7191743; doi:10.1186/s12859-020-3500-3)
Supplement: Supplementary file 1 — Additional file 1. Supplementary methods and Figures. This file contains supplementary methods and figures. [file 12859_2020_3500_MOESM1_ESM.docx]

**Supplementary method**

**Identification of circRNAs with MapSplice, CircMarker and NCLscan**

Quality control of fastq files was performed using FASTQC (<https://www.bioinformatics.babraham.ac.uk/projects/fastqc/>). Trimmomatic version 0.35 (Bolger *et al.*, 2014) was used to remove Illumina adapters and low quality bases, using a Phred score cut-off of 30 whilst discarding reads with a length below 25 bases. RNA-sequencing reads passing the quality control check were then aligned against the human genome reference hg19 with MapSplice version 2.2.0., CircMarker (release July.24.2018) and NCLscan v1.4. The gencode annotation file release v19 was used by the three predictions tools to extract the coordinates of the back-spliced junction (BSJ)-spanning reads. Furthermore, for MapSplice2 we set the following options: --min-fusion-distance 200 --filtering 1 and --min-map-len 25. For NCLscan we set the following option: span range 25 bases. For CircMarker we used the default options.

**Supplementary Figures**

**
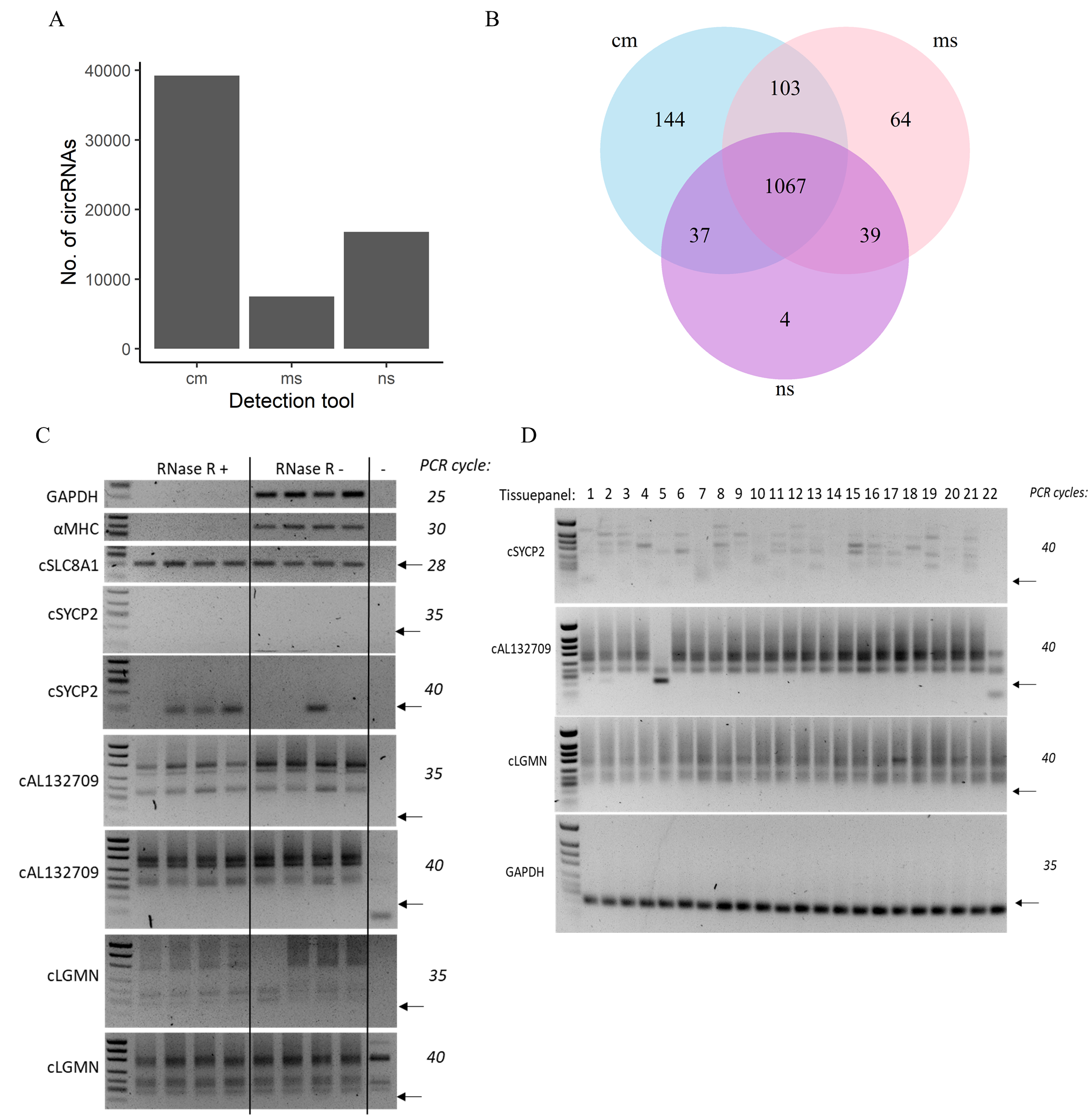
**

**Figure S1:** **circRNA prediction results.** A) Histogram showing the number of circRNAs predicted by the 3 circRNA detection tools: MapSplice2 (ms), NCLscan (ns), and CircMarker (cm). B) Venn-diagram showing the 1458 circRNAs that survived the filtering step. Cut-off used for filtering: at least 5 read counts in all samples in 1 condition (DCM, HCM, or Con). 1067 circRNAs were commonly identified in all 9 samples by all 3 prediction tools. C) Experimental validation of 3 circRNAs (i.e., cSYCP2, cAL132709, cLGMN) predicted to be highly expressed by the detection tool CircMarker in 4 human heart samples with and without RNase treatment. PCRs were run at 35 and 40 cycles and compared to the expression of cSLC8A1. Arrow indicates the predicted amplicon size. D) PCR of 3 predicted circRNAs in a human tissue panel (1. fetal liver; 2. adrenal gland; 3. stomach; 4. uterus; 5. liver; 6. skeletal muscle; 7. thyroid gland; 8. cerebellum; 9. trachea; 10. lung; 11. heart; 12. kidney; 13. whole brain; 14. spleen; 15. fetal brain; 16. placenta; 17. salivary gland; 18. prostate; 19. thymus; 20. small intestine; 21. fetal heart; 22. bone marrow). Arrows point at the predicted amplicon sizes.


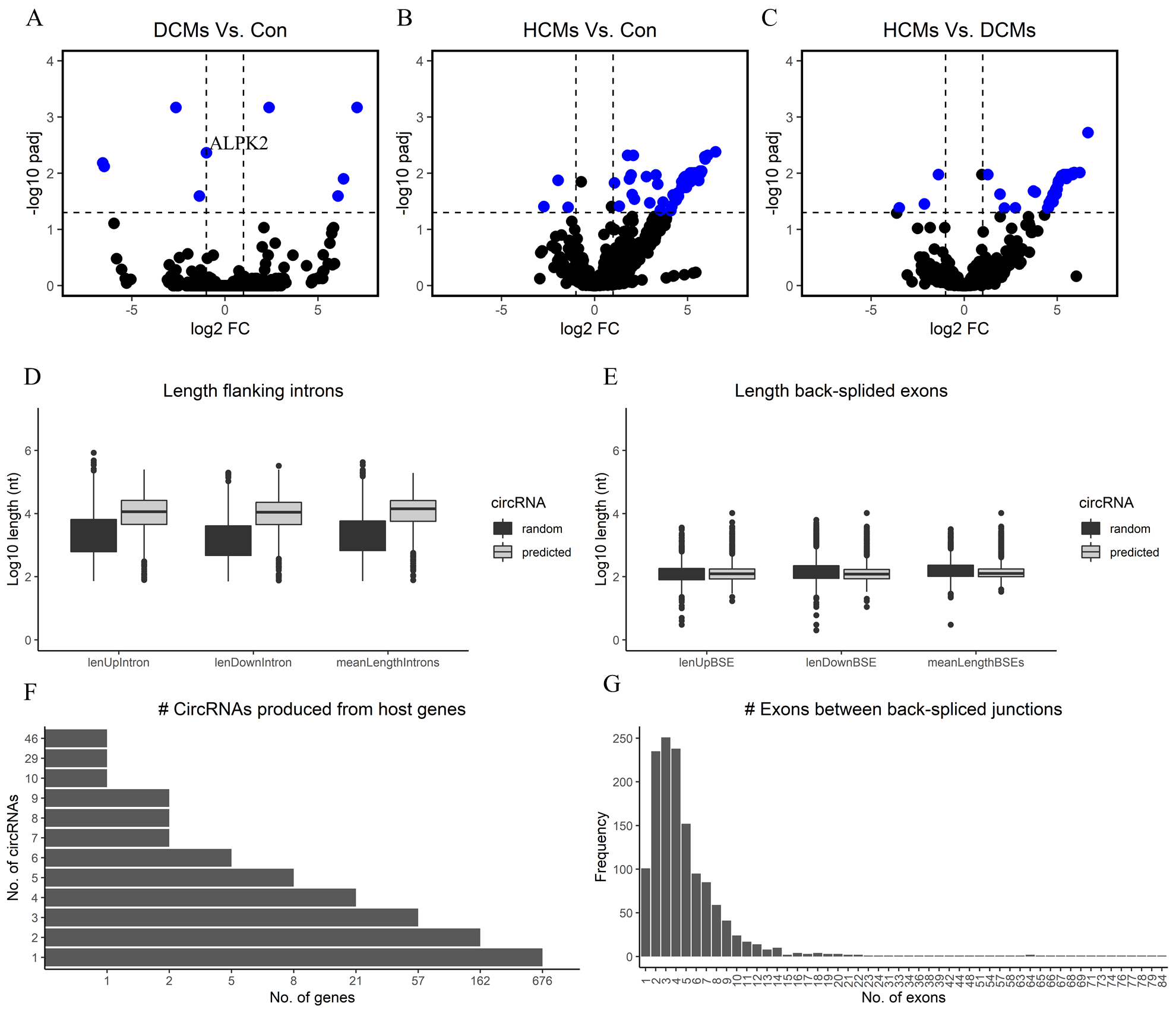


**Figure S2: Differential expression analysis and genomic context of circRNAs.** Volcano plots showing the differentially expressed circRNAs in A) DCMs Vs. Con, B) HCMs Vs. Con, C) HCMs Vs. DCMs. Differential expression analysis was performed on the subset of 1458 filtered circRNAs. The helper function for the R Bioconductor package DESeq2 was used with the following cut-offs: absolute log2FC = 1 and adjusted p-value ≤ 0.05. D) Box plots showing the length of the introns flanking the back-spliced exons of the subset of filtered circRNAs compared to the length of introns flanking an equal number of randomly generated back-spliced exons. On the X-axis, lenUpIntron is the length (nt) of the introns immediately upstream the acceptor sites. lenDownIntron: is the length (nt) of the introns immediately downstream the donor sites. meanLengthIntrons is the mean length (nt) of the introns flanking the detected back-spliced exons. E) Box plots showing the length of the back-spliced exons of the subset of filtered circRNAs compared to an equal number of randomly generated back-spliced exons. On the X-axis lenUpBSE is the length (nt) of the upstream back-spliced exons. lenDownBSE is the length (nt) of the downstream back-spliced exons. meanLengthBSEs is the mean length (nt) of the back-spliced exons. F) Histogram showing the number of circRNAs produced from the circRNA host genes. G) Histogram showing the number of exons between the predicted back-spliced junctions.


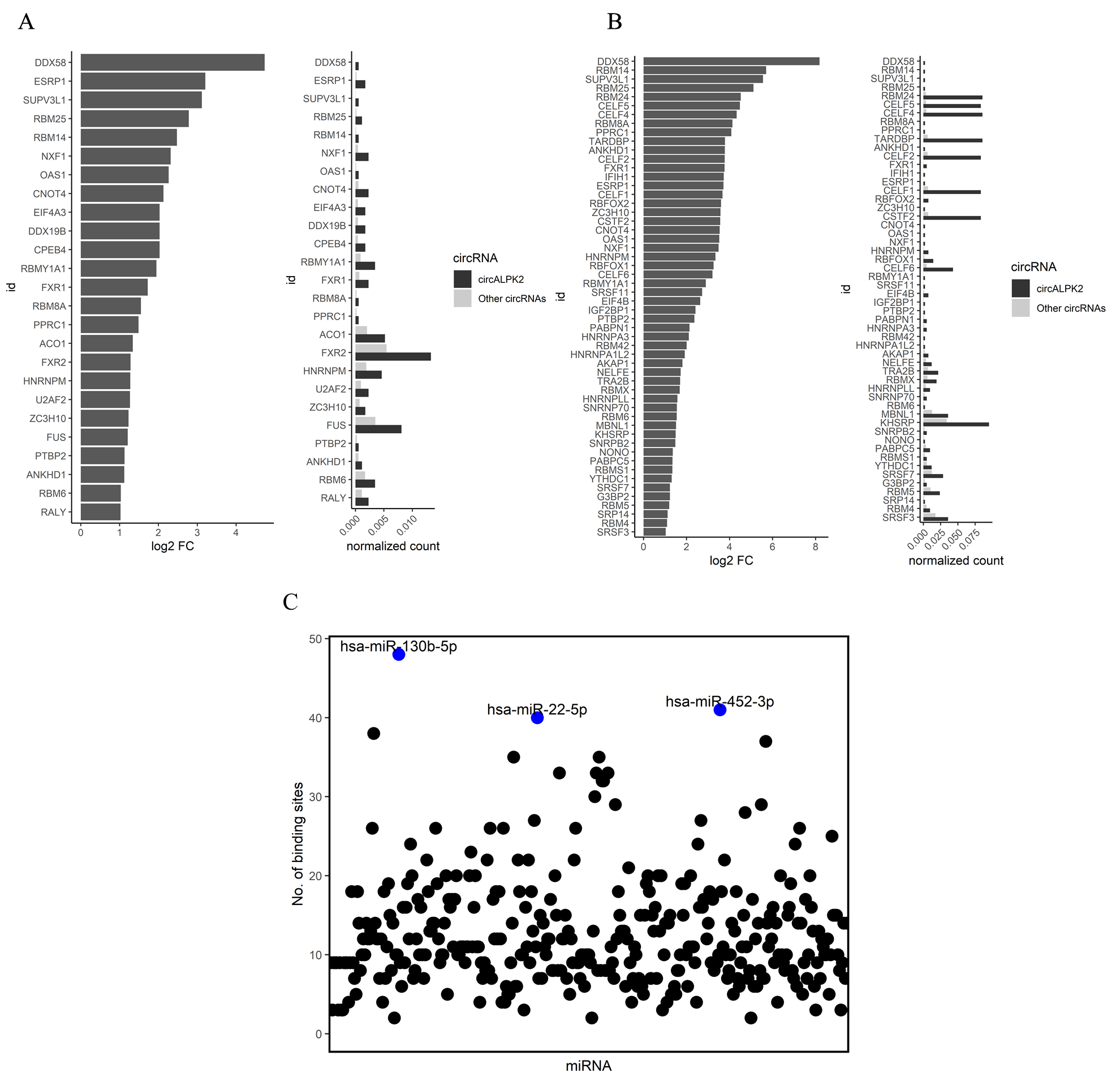


**Figure S3: RBP and miRNA binding site analysis of circALKP2.** A) Bar chart showing the log2FC (cut-off ≥ 1) and the normalized counts of the RBP motifs found in the circRNA sequence of circALPK2 compared to the remaining subset of 1457 filtered circRNAs. B) Bar chart showing the log2FC (cut-off ≥ 1) and the normalized counts of the RBP motifs found in the region flanking the predicted back-spliced junction of circALPK2 compared to the remaining subset of 1457 filtered circRNAs. For the RBP analysis, we used the default settings and added the RBM20 consensus motif ([ACU]UUCU[ACU]) in motifs.txt since the latter is not deposited in the ATtRACT database. We normalize the number of RBP motifs by the total length of the target sequences. C) Scatter plot showing the number of miRNAs binding sites found in the ALPK2 circRNA sequence. For the miRNA analysis, we used the following cut-off: ≥6 total matches between the seed region of the miRNAs and the seed site in the target sequence, of which at least 5 are canonical Watson-Crick matches and 1 can be a non-canonical match (G:U).
